# Fire and smoke real-time detection algorithm for coal mines based on improved YOLOv8s

**DOI:** 10.1371/journal.pone.0300502

**Published:** 2024-04-18

**Authors:** Derui Kong, Yinfeng Li, Manzhen Duan

**Affiliations:** School of Emergency Management and Safety Engineering, North China University of Science and Technology, Tangshan, Hebei, China; Kanazawa University, JAPAN

## Abstract

Fire and smoke detection is crucial for the safe mining of coal energy, but previous fire-smoke detection models did not strike a perfect balance between complexity and accuracy, which makes it difficult to deploy efficient fire-smoke detection in coal mines with limited computational resources. Therefore, we improve the current advanced object detection model YOLOv8s based on two core ideas: (1) we reduce the model computational complexity and ensure real-time detection by applying faster convolutions to the backbone and neck parts; (2) to strengthen the model’s detection accuracy, we integrate attention mechanisms into both the backbone and head components. In addition, we improve the model’s generalization capacity by augmenting the data. Our method has 23.0% and 26.4% fewer parameters and FLOPs (Floating-Point Operations) than YOLOv8s, which means that we have effectively reduced the computational complexity. Our model also achieves a mAP (mean Average Precision) of 91.0%, which is 2.5% higher than the baseline model. These results show that our method can improve the detection accuracy while reducing complexity, making it more suitable for real-time fire-smoke detection in resource-constrained environments.

## Introduction

Coal is an essential component of global energy consumption, accounting for a significant portion of the world’s electricity generation. With the rapid development of industry and economy, the demand for coal energy is expected to continue growing in the foreseeable future [1], so it is very important to ensure the safety of coal mining. Among the many safety risks faced by coal mining, fires are one of the most frequent and devastating occurrences [2]. These fires not only result in significant loss of life and property but also pose a severe threat to the environment and the sustainability of our energy resources [3].

As the most frequent and influential disaster in coal mines, preventing fires is of paramount importance to protect both human lives and energy resources. This is particularly crucial in countries where coal is a major source of energy, such as China and India [4]. To ensure the safety of miners and prevent the devastating consequences of coal mine fires, it is necessary to implement effective preventive measures [5].

At present, the detection of coal mine fire-smoke mainly relies on manual inspection and traditional fire detection equipment. These methods have the following problems: First, the process of manually inspecting requires a significant amount of time and effort, and it is easily affected by environmental conditions and human factors, resulting in low detection efficiency and poor accuracy. Second, traditional fire detection is based on various sensors, using sensors to detect the temperature and smoke concentration, and alarm when a certain threshold is reached. However, this method is easily affected by various interference factors, and the threshold also depends on artificial specification. Computer vision-based fire-smoke detection systems can address these issues effectively, reducing labor costs and enhancing detection accuracy.

Firstly, Computer vision-based fire-smoke detection systems offer the potential for automated detection, eliminating the need for continuous human monitoring and reducing labor costs. By using advanced algorithms, these systems can analyze visual data in real-time and identify signs of fire and smoke, enabling rapid response. Additionally, computer vision-based systems can enhance detection accuracy. They can detect fire and smoke with a high degree of sensitivity, even in challenging environments where traditional detection methods may struggle. This increased accuracy can lead to earlier detection, allowing for quicker intervention and potentially preventing the spread of fires.

One of the key components of these systems is the real-time detection algorithm, because the algorithm determines detection accuracy and speed [6]. The use of computer vision algorithms, has significantly improved the accuracy and efficiency of coal mine fire detection [7]. This is essential for detecting potential hazards and ensuring the timely response to mitigate any potential damage.

## Literature review

The early algorithms for fire-smoke detection using computer vision relied on extracting static and dynamic features of fire-smoke [8]. They mainly extracted the color, shape, and texture features of smoke, then fused them with multiple features and set a manual threshold, and finally identified the smoke [9, 10]. In this way, the detection results are subjective with low accuracy. In addition, smoke can also be detected by observing the spatial wavelet transform of the current image and the background image [11], but it is easily affected by smoke-like objects, increasing the possibility of false detection. To further improve the accuracy, Cheng et al. extracted smoke features based on the different color distributions of smoke in R, G, and B channels [12], Tian et al. extracted dynamic features of smoke by using background subtraction and optical flow methods [13]. However, whether based on static features, dynamic features, or a combination of static and dynamic features, the feature extraction operators are basically obtained by experience, subjective, and not efficient enough in detection.

Using deep learning for fire-smoke detection has become the main trend due to the rapid progress of neural network, usually in two ways: the first method is to use a classification network to identify the image. The classic classification network Convolutional Neural Network (CNN) has achieved good results in fire detection [14]. After that, many scholars improved CNN to make it more suitable for smoke and flame recognition. Deep Normalization CNN (DNCNN) [15] replaces the traditional convolution layer of CNN with a normalization convolution layer, which has the potential to enhance the efficiency of training procedures and optimize the effectiveness of smoke detection. In addition, image denoising is an important issue in fire-smoke detection. Many fires occur at night and are accompanied by a lot of dust. DenseNet [16] can recover clear images from noisy images. The method of using DenseNet to complete flame recognition is simple, reliable, and low-cost in hardware equipment. As long as there is smoke or flame on the image, the alarm mechanism is triggered [17]. But the classification network can only classify the target, and cannot locate the target. This makes it impossible for computer to determine the location of the fire only from picture. The second method is to apply an object detection algorithm to fire and smoke images, which can solve the problem of target location. Being a representative one-stage detection network, Faster R-CNN [18] demonstrates exceptional accuracy. Using Faster R-CNN as the flame detection network can achieve good detection results [19]. However, its size is too large to deploy, and the algorithm has poor real-time performance, so it cannot cope with the daily fire-smoke detection work. Therefore, scholars have done a lot of work on network compression, such as using lightweight convolutional neural network MobileNetv3 [20] to reconstruct the backbone of object detection algorithm SSD [21], which complexity is already very low, effectively reducing the model parameters [22], which provides us with ideas for improvement. However, reducing only the complexity of the model is likely to reduce the detection accuracy, so it is necessary to introduce some modules that can improve the accuracy for balance.

**Fig 1:**
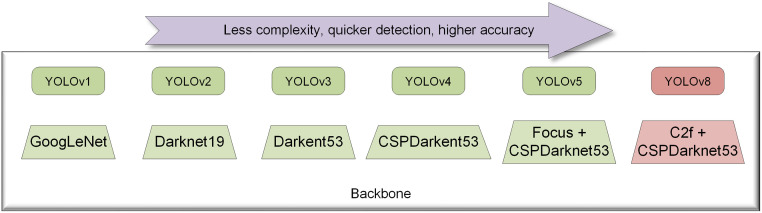
Diagram of YOLO evolution.

You Only Look Once (YOLO) has become popular as a computer vision-based CNN algorithm that requires less hardware and detects faster. The YOLO family has produced a lot of versions, which are typical algorithms of single-stage detection. As YOLO iterates, its accuracy and complexity are optimized. As can be seen in Fig 1, each update is accompanied by a major change in the backbone. YOLOv1 [23] algorithm uses a single GoogLeNet with no inception modules to realize the whole process from graphic input, candidate box generation to final predicting category and bounding box regression parameters. YOLOv2 [24] enhances accuracy through the incorporation of batch normalization, anchor boxes. And by replacing the classification network with Darknet-19, the overall convolution operation is less than that in YOLOv1, which reduces the amount of computation. YOLOv3 [25] further enhances the model performance by using Darkent53 as backbone. YOLOv4 [26] integrates the CSP structure into Darknet53 and generates a new backbone network, CSPDarkent53, which can effectively enhance the learning ability of the network. Meanwhile, it introduces a new anchor-free detection head and a new loss function to reduce detection time. YOLOv5 adds a focus framework to backbone to speed up training. In addition, the CSP module is applied to the neck structure. Before YOLOv8, YOLOv5 is a more widely used target detection model. Since YOLOv6 and v7 are less used and the effect is not as good as YOLOv5 in some respects, we will not go into details here. For the YOLO algorithm, researchers have also made many additional improvements, which give us some very meaningful references. Wang et al. used depth-wise separable convolution to replace the ordinary convolution in the YOLOv4 network to achieve model lightweighting [27], but model lightweighting often leads to a decrease in detection accuracy. Zhang et al. introduced ECA-Net [28] attention mechanism to enhance the feature extraction ability of the YOLOv5 model [29], but the improvement of accuracy often accompanies the increase of model’s parameters [30], which cannot guarantee the completion of real-time detection tasks. Therefore, how to make a good trade-off between accuracy and complexity is a big challenge. With the rapid iteration of the YOLO series, YOLOv8 makes further improvements on the basis of YOLOv5. C2f module is introduced into DARKnet53 as the backbone, and the detection accuracy is greatly improved. Therefore, it is more appropriate to choose YOLOv8 as the baseline model for further improvement.

The smoke and fire detection in coal mine environment poses high requirements on the model’s capacity to precisely and swiftly detect [31]. Because, in the early stage of fire, the targets of smoke or flame are often small, and the dust, light, and other objects similar to smoke and flame in the coal mine environment will interfere with the detection. If the early flame is not intervened, the fire will spread rapidly [32]. Although YOLOv8 can detect smoke and fire in coal mine in real time in terms of detection speed, as a one-stage object detection algorithm, but the model’s detection precision and sensitivity to small targets are hard to satisfy the practical needs. To effectively improve accuracy and simplify complexity, we propose an improved YOLOv8 algorithm for real-time smoke and fire detection in coal mine, with the following steps: First, we add interference factors to the dataset, such as Mosaic data enhancement, to improve the model’s generalization ability. Second, in order to compensate for the computational cost problem caused by subsequent improvements, we use Slim-neck [33] to reconstruct the neck of YOLOv8 and Partial Convolution [34] to enhance the C2f component within the backbone by minimizing the parameters. Further, we introduce Efficient Multi-Scale Attention [35] mechanism into the backbone of YOLOv8, making the model focus on small target information, reducing false positives and negatives in detection. Finally, we use Dynamic Head [36], which is designed for object detection under different light or climate environments, to improve the original head, suppress the interference of background noise, and avoid missed detection and false alarms. The various key gaps in existing work which are going to address through this paper are shown in Table 1.

**Table 1:** Existing works’ key gaps and our solution.

Key gaps	Solution
High Complexity	Lighter by GSConv and PConv
low Accuracy	Introducing Attention Module
Banlance between Accuracy and Complexity	Dynamic Detection Head
Poor generalization ability	Data Mosaic

The contributions of our research are as follows:

- By introducing GSConv and PConv, we significantly reduce the complexity of YOLOv8s.
- By introducing attention mechanism in the backbone and use Dynamic head, we effectively improve the accuracy of the model.
- Our improved model achieves a good balance between precision and complexity, and can operate reliably in coal mine environment.

## Materials and methods

### Data manipulation

#### Data collection and labeling

In order to make the final model perform well in the task of fire-smoke detection in coal mine, we selected the WildfireSmokeDataset from EXTREM MART, which includes 737 labeled wild fire-smoke images. Because the smoke targets in the wild are small and the wild environment is similar to coal mine [37]. In addition, we also select the open_fire dataset from Github to make the fire and smoke scenarios more diverse, using 1,446 labeled fire-smoke images from it. The dataset includes images of vehicles and buildings that are on fire, which is common in coal mines and cities. The final integrated dataset includes 2183 daytime and nighttime wild, building, and vehicle fire-smoke images, named as smoke_fire_1, which has been made public on Robowflow.

The original dataset has some labels in VOC format, and we use labeling software to convert the labels to YOLO format and change all the image pixels uniformly to 640 × 640, to adapt to the subsequent model training. We also manually inspected the labeled dataset to ensure its accuracy as much as possible.

#### Data enhancement

**Fig 2:**
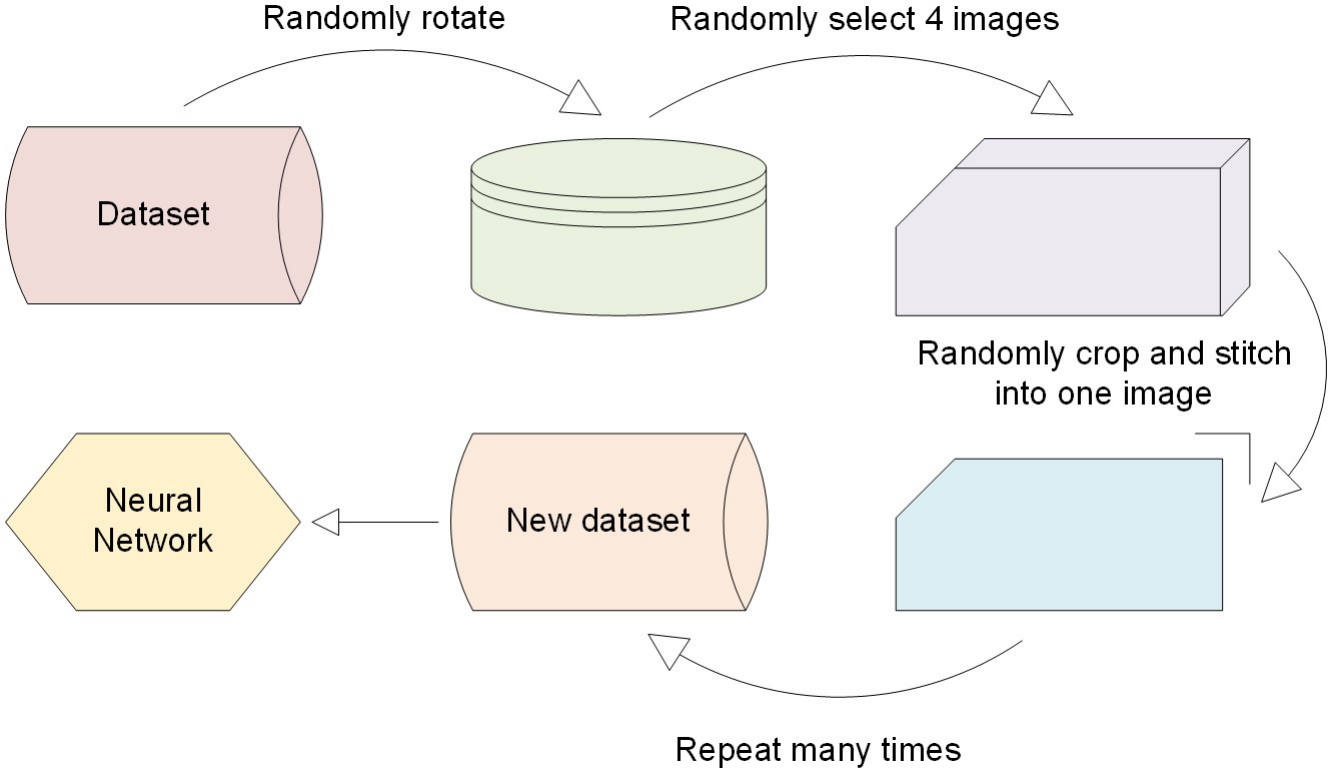
Data enhancement process.

A large amount of data is often essential for a good-performing object detection model. However, collecting new data is costly and time-consuming, so data enhancement is very useful. Data enhancement techniques can create new data by computer. such as scaling, translation, rotation, and color transformation towards original images. We combine two data enhancement methods, the first one is to make the image rotate randomly at multiple angles, such as 90° and 270°. The second one is Mosiac, which mainly cuts and scales four images randomly, then arranges and stitches them into a 640 × 640 pixel image randomly. Fig 2 illustrates the procedure of data enhancement.

**Fig 3:**
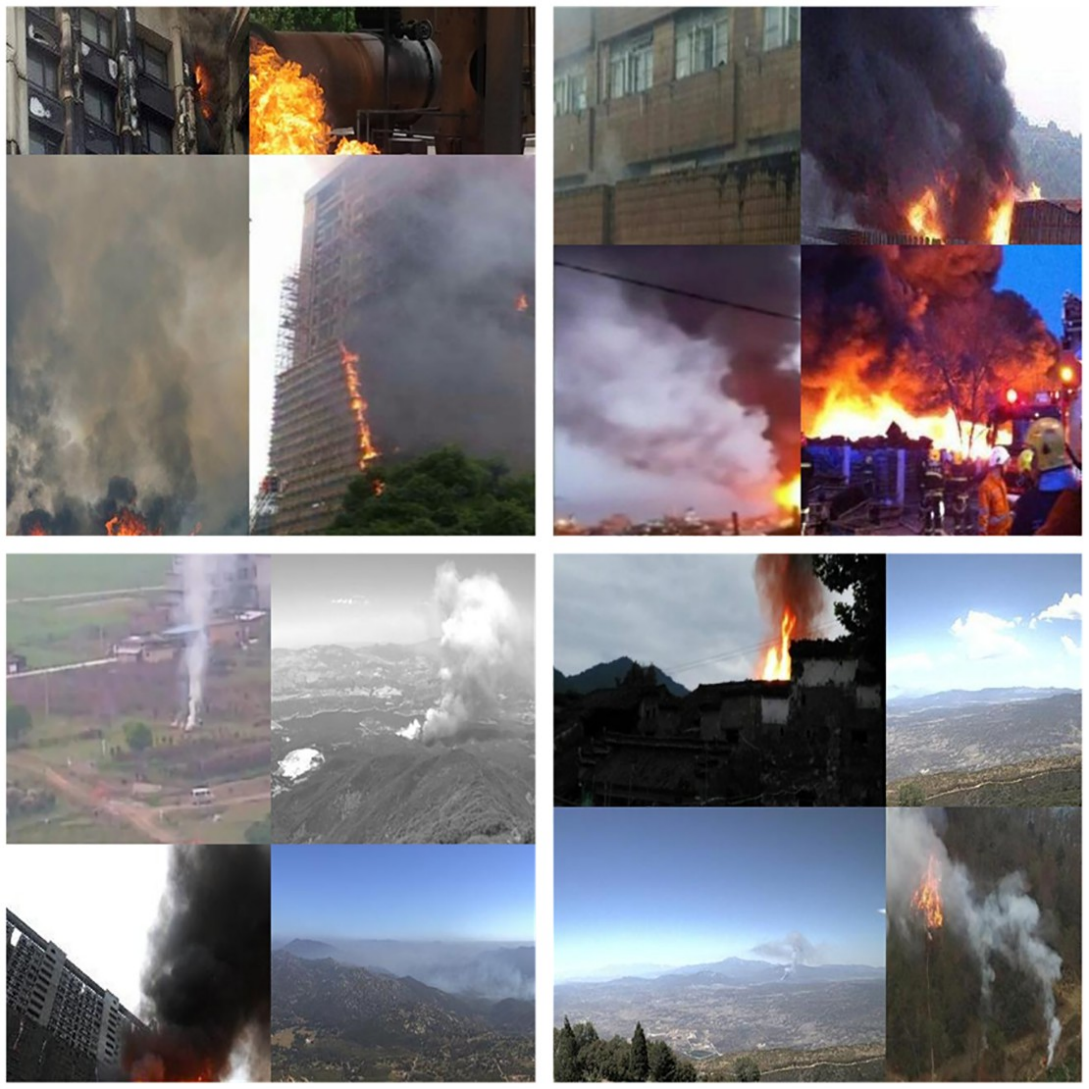
Diagram of part of the data enhancement.

Randomly rotating the image can enhance the model’s capability to detect targets with different angles and orientations, and reduce the model’s sensitivity to the background and noise of the image. Mosiac’s random cropping and scaling of the image can improve the model’s capability to detect targets with different scales and background conditions. The coal mine area is generally large, and if a fire occurs, its distribution may be scattered and far away from the camera. Using an augmented dataset for training can make model effectively detect these small targets in the distance. Some images after data enhancement are shown in Fig 3. There are a total of 3,711 images included in the final dataset.

### Baseline model YOLOv8s

YOLOv8 primarily consists of three components: backbone, neck, and head, which form the fundamental framework. We will improve the backbone and neck parts for lightweighting, and introduce attention mechanisms to the backbone and head parts for enhancing accuracy.

In YOLOv8, CBS refers to the Conv-BN-SiLu convolution module, which comprises a convolutional layer, a layer for batch normalization, and an activation function (Sigmoid Linear Unit). C2f module is a convolution module composed of multiple CBS. SPPF refers to the Spatial Pyramid Pooling-Fast [38], which serves as a module for extracting features to solve defects caused by different sizes of input images. Split refers to the operation of splitting the input tensor along a specified dimension into multiple tensors. Upsample refers to the upsampling operation, which enlarges the low-resolution image or feature map to high-resolution, increasing the model’s ability to recognize details. Concat refers to the fusion operation, which fuses the features extracted by multiple convolutional feature extraction frameworks.

The previous sota model YOLOv5, which can be used for real-time detection, is different from the current YOLOv8 in the following parts: First, the convolution of YOLOv8 uses C2f module, while YOLOv5 uses C3 module, which is slightly more complex than C2f. Second, YOLOv8 removes part of the CBS module in YOLOv5, making it more lightweight. Finally, the YOLOv8 uses Decoupled Head instead of the Coupled Head of YOLOv5, which improves accuracy to a certain extent. To sum up, YOLOv8 is stronger than YOLOv5 in terms of complexity and accuracy, so this paper chooses YOLOv8 as the baseline model.

YOLOv8 has five models with the same structure but different sizes, which can handle different object detection tasks, namely YOLOv8n, YOLOv8s, YOLOv8m, YOLOv8l, and YOLOv8x. The detection accuracy, detection time, number of parameters, and FLOPs of the five models n, s, m, l, and x increase one by one. Compared with m, l, and x, YOLOv8s has the least increase in the parameters and FLOPs (Floating-Point Operations), while the mAP (mean average precision) is improved most significantly. Therefore, for the real-time detection task of fire-smoke in coal mine, YOLOv8s is the most suitable baseline model.

### The process of improvement

#### Slim-neck by GSConv

**Fig 4:**
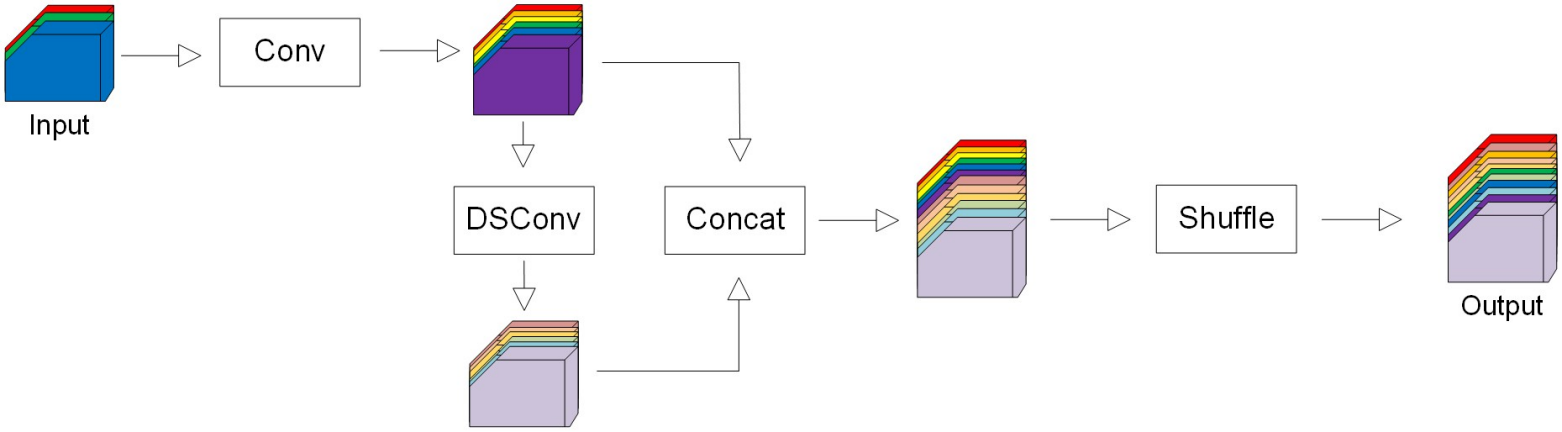
Structure illustration of GSConv.

**Fig 5:**
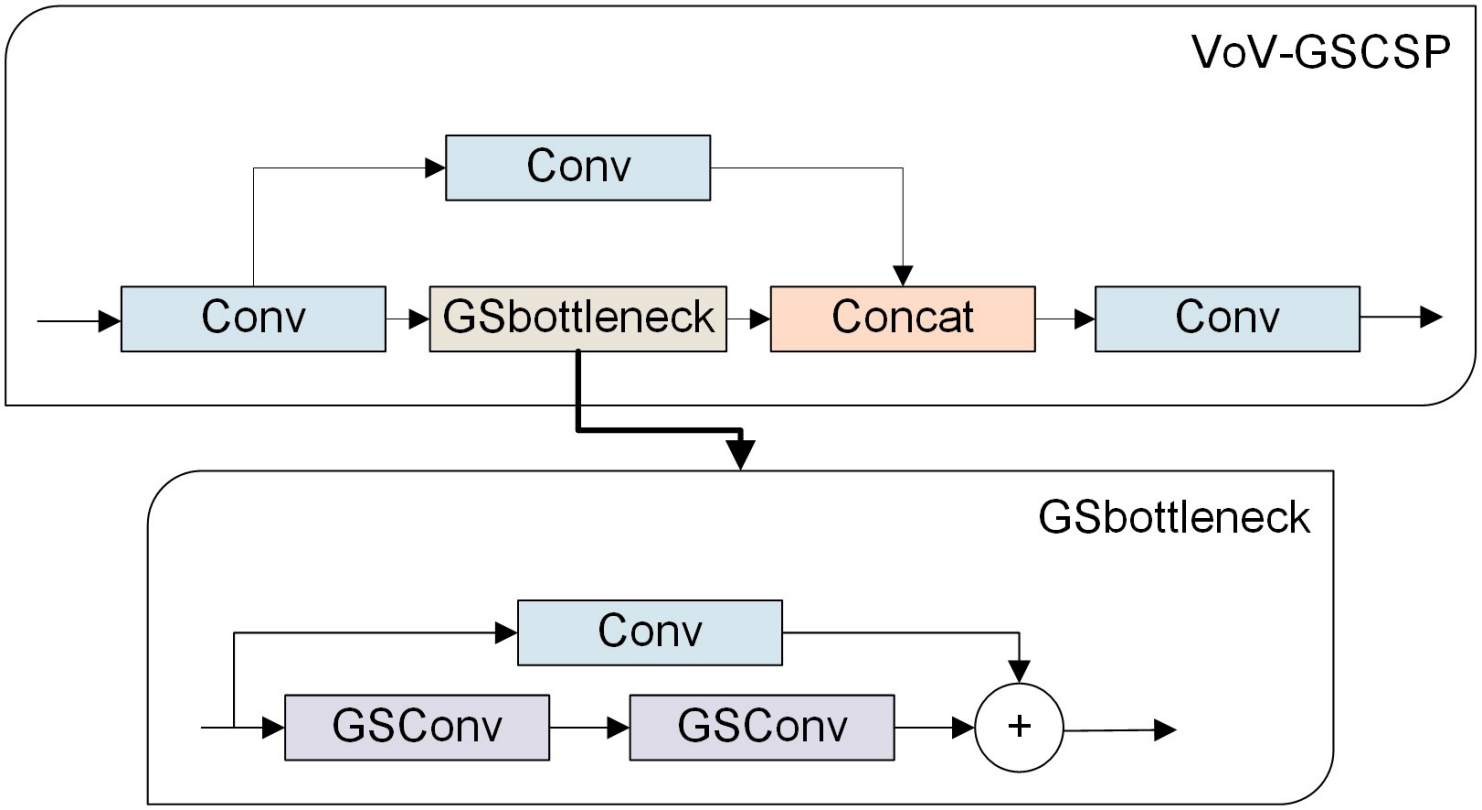
Structure illustration of VoV-GSCSP.

Large-scale models are generally difficult to deploy on computers with poor configurations, especially in coal mines that have relatively backward equipment. In order to make the improved model widely applicable, the first step is to decrease the complexity of model as much as possible without compromising accuracy. GSConv is a convolution composed of Standard Convolution (Conv), Depth-wise Separable Convolution (DSConv), and data shuffling module (shuffle). DSConv separates a complete convolution operation into two steps, and the number of parameters is greatly reduced [39]. GSConv and Conv-BN-SiLu are both lightweight convolution methods, but GSConv can maintain similar feature extraction and fusion capabilities as standard convolution while reducing computational complexity. The architecture of GSConv is depicted in Fig 4, where shuffle penetrates the information generated by Conv into the processing results of DSConv. Conv-BN-SiLu has its framework C2f, similarly, there is also a framework based on GSConv, which is a cross-stage partial network module, called VoV-GSCSP, whose structure is shown in Fig 5.

The enhanced non-linear expression ability of DSConv and shuffle combination can improve the accuracy while keeping the model lightweight. Using GSConv in each stage of the model will lead to an increase in the network layer’s depth, hindering the flow of data and significantly prolonging the prediction time. To mitigate this issue, it is recommended to exclusively implement GSConv in the neck, enhancing the channel dimension of the feature map without compromising efficiency. That is, Slim-neck. GSConv is suitable for processing the concatenated feature maps in the neck, because it produces less redundant and duplicate information, and does not need to compress the feature maps.

The changes to the neck of YOLOv8s include the following two operations: replacing CSB with GSConv and replacing C2f module with VoV-GSCSP.

#### C2f-faster by PConv

**Fig 6:**
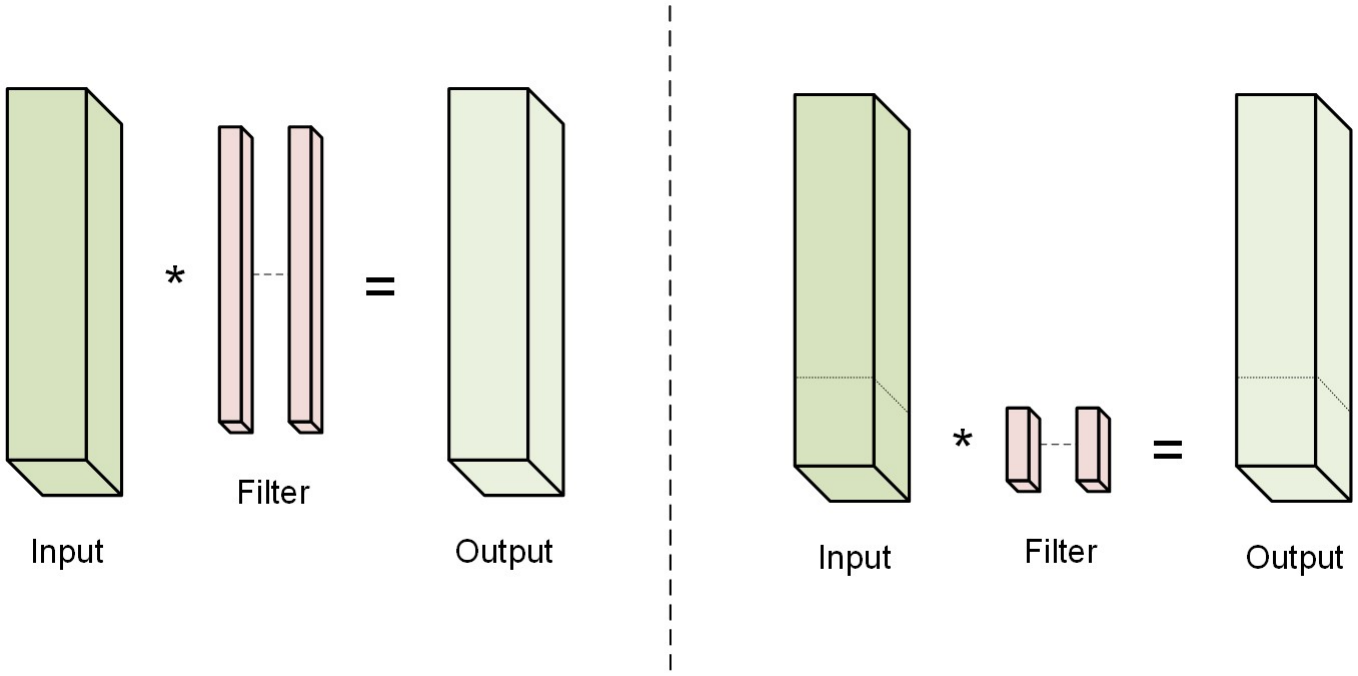
Convolution versus Partial Convolution. The left side of the picture shows the process of Standard Convolution, the right side shows the process of Partial Convolution.

In order to ensure that our model is lighter than the baseline model, and leave some room for subsequent improvements for accuracy, we need to further improve the backbone part for lightweighting. To achieve a more lightweight network, while minimizing the increase in neural network latency, we introduce Partial Convolution (PConv) in the C2f module, which differs from standard convolution in that PConv uses traditional convolution only on a portion of the input channels to extract spatial characteristics, while leaving the rest of the channels unmodified, as shown in Fig 6.

PConv accelerates prediction by reducing redundant computation and memory access, and can improve FLOPS (Floating-Point Operations per Second) while reducing FLOPs (Floating-Point Operations), effectively reducing complexity, but it will cause some degree of accuracy degradation. The improvement for the C2f module in the backbone part is to replace the Conv with PConv, and the improved C2f is called C2f-faster.

#### Introduce attention module to backbone

Attention mechanism can help the model better handle complex scenes and small targets, reduce the occurrence of false positives and false negatives. The structure of Efficient Multi-Scale Attention Module (EMA) consists of two parallel sub-networks, one is a 1x1 branch, which is used to capture global information in different spatial directions and perform channel-wise interaction; the other is a 3x3 branch, which is used to capture local multi-scale spatial information. The advantage of EMA is that it does not need to reduce the channel dimension, retaining the information of each channel, and using grouping and multi-scale structure, effectively establishing short-range and long-range dependencies. In other words, it can make the model better focus on the characteristics of smoke and flame, thereby improving the model’s ability to distinguish between background and target, and avoid both missed detections and false alarms.

EMA is inserted after the C2f-faster of the backbone, that is, added to the part that extracts the image features. By adding the attention mechanism, the model has the capability to prioritize the extraction of small target information, suppress the interference caused by background noise, and intensify the emphasis on the intended target, and thus improve the recognition and localization accuracy of small flames and complex smoke targets in coal mine.

#### Dynamic Head with attention module

An excellent object detection head should possess three key abilities as follows: First, the head should have scale-awareness ability, because multiple objects with large scale differences often coexist in an image. Second, the head should have spatial-awareness ability, because objects usually present significantly different shapes under different viewpoints. Third, the head needs to have task-awareness ability, because different detection objects have completely different object features and constraints. In previous research, most of the improvements to the head only target one of the above aspects.

**Fig 7:**
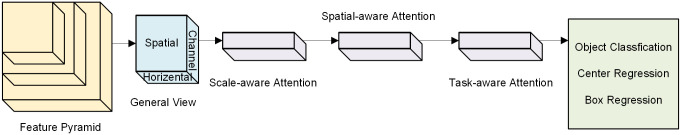
An illustration of the Dynamic Head approach.

The Dynamic Head (DyHead) introduced in this paper combines the improvements for scale-awareness, spatial-awareness, and task-awareness in the head part. Fig 7 illustrates the approach of implementing attention mechanisms on individual feature dimensions as the enhancement technique. The horizontal dimension utilizes the scale-aware attention module, while the spatial dimension incorporates the spatial-aware attention module. On the other hand, the channel dimension incorporates the task-aware attention module.

The scale-aware attention module can learn the relative importance of features at different semantic levels according to the scale of each object, improving the ability to recognize objects of different sizes. The spatial-aware attention module can adaptively aggregate multiple feature layers to learn more discriminative feature representations, which allows the model to recognize different shapes of smoke and flame. The task-aware attention module can make different feature channels prefer different detection tasks according to different convolutional kernels, which allows the model to sense different colors and thicknesses of smoke. Therefore, DyHead can dynamically adjust the importance and fusion mode of features to adapt to different inputs and outputs, which will minimize the issue of false positives and negatives in detection. Additionally, each attention module of DyHead only performs attention calculation on one dimension of the feature tensor, so the computational complexity will not increase.

### An improved smoke and fire detection model based on YOLOv8s

**Fig 8:**
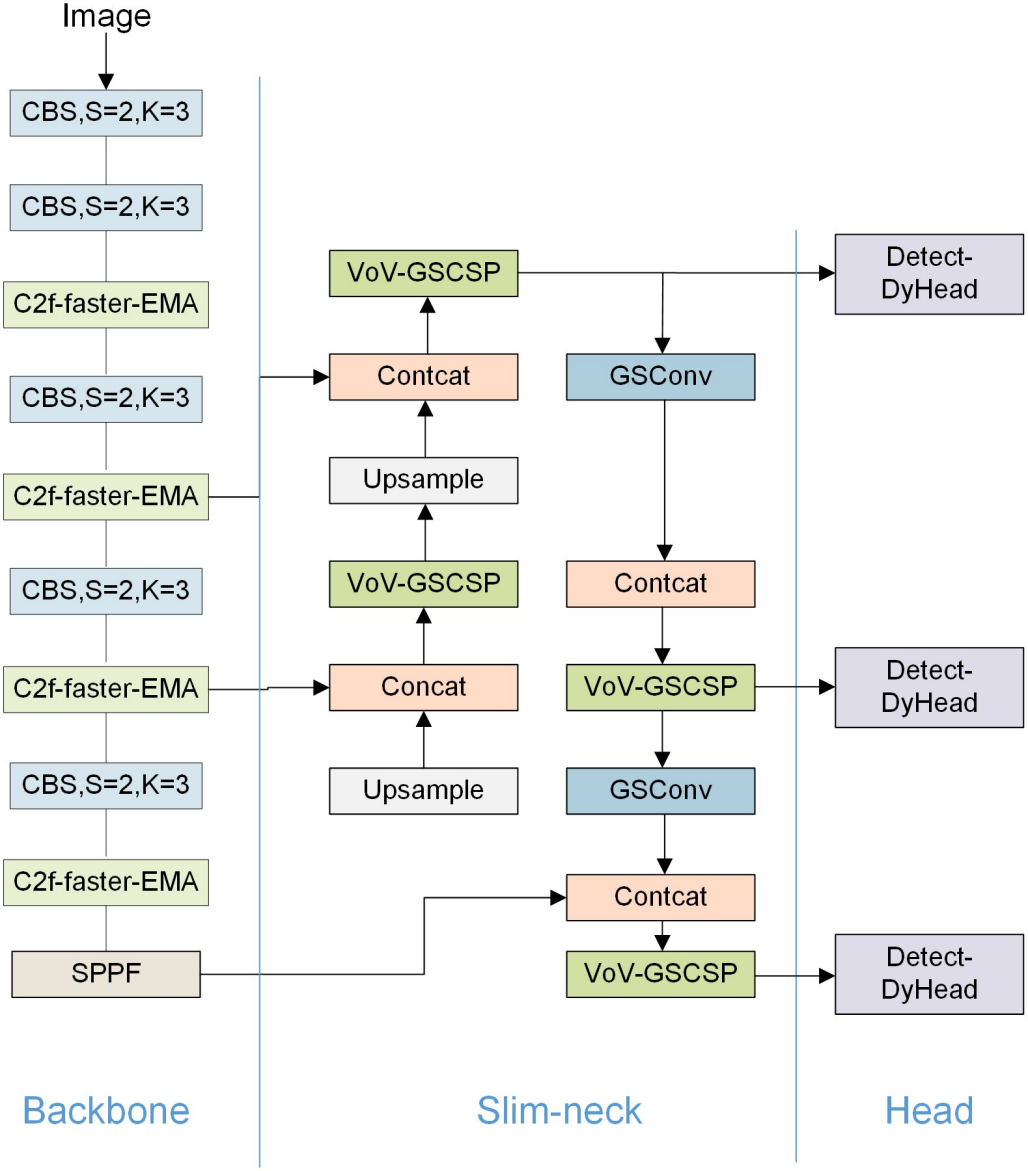
The ultimate revised network configuration.

Ultimately, We improved the overall network structure of the model based on YOLOv8s, as illustrated in Fig 8. Backbone part: introduced the EMA module, which enhances the model’s capacity to detect minor fire-smoke occurrences, but increases the complexity. To cope with the rising complexity, PConv is used to replace Conv in C2f, which significantly reduces complexity and speeds up the computation; neck part: GSConv and VoV-GSCSP are used to replace CBS and C2f modules, GSConv is more lightweight while maintaining the same feature extraction ability, making YOLOv8s’s neck slimmer; head part: introduced DyHead to replace the original detection head, which introduces three kinds of attention mechanisms on different dimensions to avoid missed detections and false alarms as much as possible.

### Assessment of the model performance

To enhance the assessment of the model’s performance and validate its efficacy of each improvement point, the evaluation indicators selected for the experimental results mainly include: Precision (P), Recall (R), Average Precision (AP), mean Average Precision (mAP), F1-score, Parameter Size(Params), FLOPs (Floating-Point Operations), and single image detection time (Time). Params and FLOPs could be seen as computation complexity. P, R, mAP and F1-score are collectively referred to as accuracy. Eqs (1), (2), 3), (4) and (5) showcase the computation formulas for P, R, AP, mAP and F1-score respectively.

$$\begin{array}{c} P=TP/(TP+FP), \end{array}$$ (1)

$$\begin{array}{c} R=TP/(TP+FN), \end{array}$$ (2)

$$\begin{array}{c} \text{AP}=\int_{0}^{1}P\left(R\right)dR, \end{array}$$ (3)

$$\begin{array}{c} \text{mAP}=\sum_{i=1}^{N}AP_{i}/N, \end{array}$$ (4)

$$\begin{array}{c} F1=2PR/(P+R), \end{array}$$ (5)

In these equations: True Positives (TP) refers to the model prediction results and the ground truth are both fire-smoke, that is, correctly detected fire-smoke; False Positives (FP) refers to the prediction result is fire-smoke, but the ground truth is background, that is, wrongly detected background as fire-smoke; False Negatives (FN) refers to the prediction result is background, but the ground truth is fire-smoke, that is, missed fire-smoke; the model’s all detections includes both fire and smoke, which can be denoted as “TP+FP”; “TP+FN” represents the overall count of actual instances of fire-smoke in an image; AP value indicates the measure of the area beneath the P-R curve; mAP value is achieved by taking the average of the AP values for all detected classes. “N” in the equation of mAP represents the total number of classes detected; Precision and Recall are contradictory measures. When Precision is high, Recall is often low. When Precision is low, Recall is often high. In order to be able to integrate these two metrics, F1-score is proposed to better understand model performance; the algorithm detection effect and the recognition accuracy are better and higher when the mAP value is higher in this study.

## Results

The whole process of our experiment was as follows: First, the well-organized dataset was partitioned into three segments: the training set, validation set, and test set, following a distribution ratio of 7:2:1. Then, the model was trained using the training set, and tuned using the validation set, so that the model was optimized in the correct direction. Afterwards, the model underwent evaluation by utilizing the test set, and successfully obtained detection results. Subsequently, a comparison was conducted between the obtained detection results and the actual test set labels, yielding performance indicators such as accuracy for the model.

**Fig 9:**
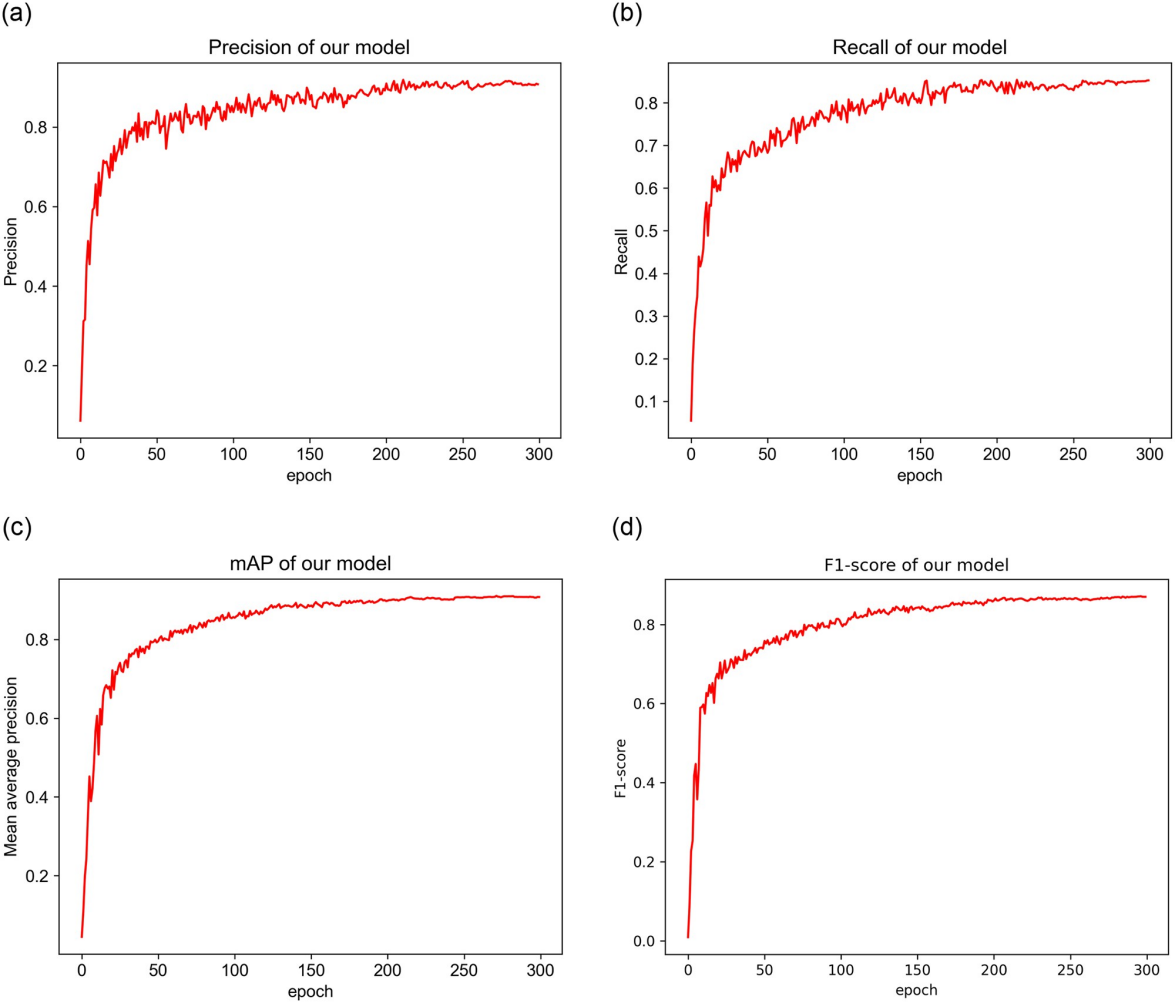
Curves illustrating the progress of training. a: Precision; b: Recall; c: mean Average Precision; d: F1-score.

The training parameters for the model were as follows: a learning rate of 0.01, momentum of 0.937, weight decay of 0.0005, a batch size of 16, and a total of 300 epochs. To optimize the trained model, the Adaptive Momentum Estimation (Adam) algorithm was used. For the experiment, a Ryzen 7 5800H CPU and a NVIDIA GeForce RTX 3060 Laptop GPU were employed. The Pytorch deep learning framework, alongside Python, was utilized for implementing the experiment. Additionally, the CUDA 11.7.1 parallel computing framework and CUDNN 8.5 deep neural network acceleration library were integrated. Fig 9 illustrates the P, R, mAP and F1-score values throughout each epoch of the training process.

Fig 9 illustrates that the model’s Precision and Recall demonstrate a gradual increment as the number of epochs increases, but the values were not stable enough. The model learned very fast during the initial phase of training, so the mAP and F1-score growth rate was faster at this stage. After 50 epochs, the increase of mAP and F1-score value tended to be stable. The mAP and F1-score curve was almost stable when the epoch reached 150 times.

Even if the fire-smoke images had different characteristics of shape, color, and texture, our model could effectively detect fire-smoke. The mAP of the best model is 91.0%, the F1-score is 0.878, the Precision is 90.6%, and the Recall is 85.1%, demonstrating that our model has a very low probability of false positives and negatives.

The performance of the enhanced model was assessed by conducting a comparative analysis with several other object detection models in a consistent experimental setting. The obtained results, as depicted in Table 2, showcase the outcome of our experiments. By examining the information provided in the table, it becomes apparent that the enhanced model surpasses models that attain good trade-offs between complexity and precision, such as YOLOv6-Tiny [40], and YOLOv7-Tiny [41]. Moreover, our model has a 2.8 percent mAP improvement over the baseline model. Compared with other models, our model is more capable of performing accurate real-time fire-smoke detection tasks in coal mines.

**Table 2:** The results of smoke and fire detection based on different models.

Model	mAP	Precision	Recall	F1-score
*Faster* − *RCNN*	75.4%	76.5%	68.1%	0.721
*YOLOv*5*s*	83.8%	86.5%	78.3%	0.821
*YOLOv*5*m*	88.1%	89.6%	76.9%	0.827
*YOLOv*6 − *Tiny*	86.9%	85.8%	86.1%	0.859
*YOLOv*7 − *Tiny*	87.6%	88.4%	81.3%	0.847
*YOLOv*8*s*	88.5%	89.1%	83.1%	0.881
*Ourmodel*	91.0%	90.6%	85.1%	0.878

To validate the real-time capabilities of our method, we calculated the Frames Per Second (FPS) and single image detection time (Time) based on the detection time of each single image for each model, as shown in Table 3.

**Table 3:** The real-time capabilities of different models on fire-smoke detection.

	Fast-er-RCNN	YOLOv5s	YOLOv5m	YOLOv6-Tiny	YOLOv7-Tiny	Our model
*Time*(*ms*)	37.4	23.5	39.8	17.9	16.9	10.0
*FPS*	27	43	25	56	59	100

As can be seen from the table, if other factors are not considered, theoretically our model’s single image detection time is lowest and detection speed can reach 100 FPS, far exceeding other models’ speed and the 30 FPS required for real-time detection. Therefore, the application of our model in coal mines enables efficient detection of fire and smoke in real-time.

In order to verify The improved model’s requirements for computational resources, we conducted statistics on the number of parameters (Params) and Floating-Point Operations (FLOPs) of each model, as shown in Table 4.

**Table 4:** The complexity of different models.

	YOLOv5s	YOLOv5m	YOLOv6-Tiny	YOLOv7-Tiny	YOLOv8s	Our model
*Params*(*Million*)	9.1	21.07	9.7	6.07	11.1	8.6
*FLOPs*(*Giga*)	15.8	47.9	24.8	13.32	28.4	20.9

As can be seen from the table, our model has low requirements for computational resources compared to other common models. Moreover, compared to baseline, our method has 23.0% and 26.4% fewer Params and FLOPs. Therefore, in the current situation, our model performs better in resource-constrained environments typically found in many mining operations.

## Discussion

### Effectiveness of detecting fire

**Fig 10:**
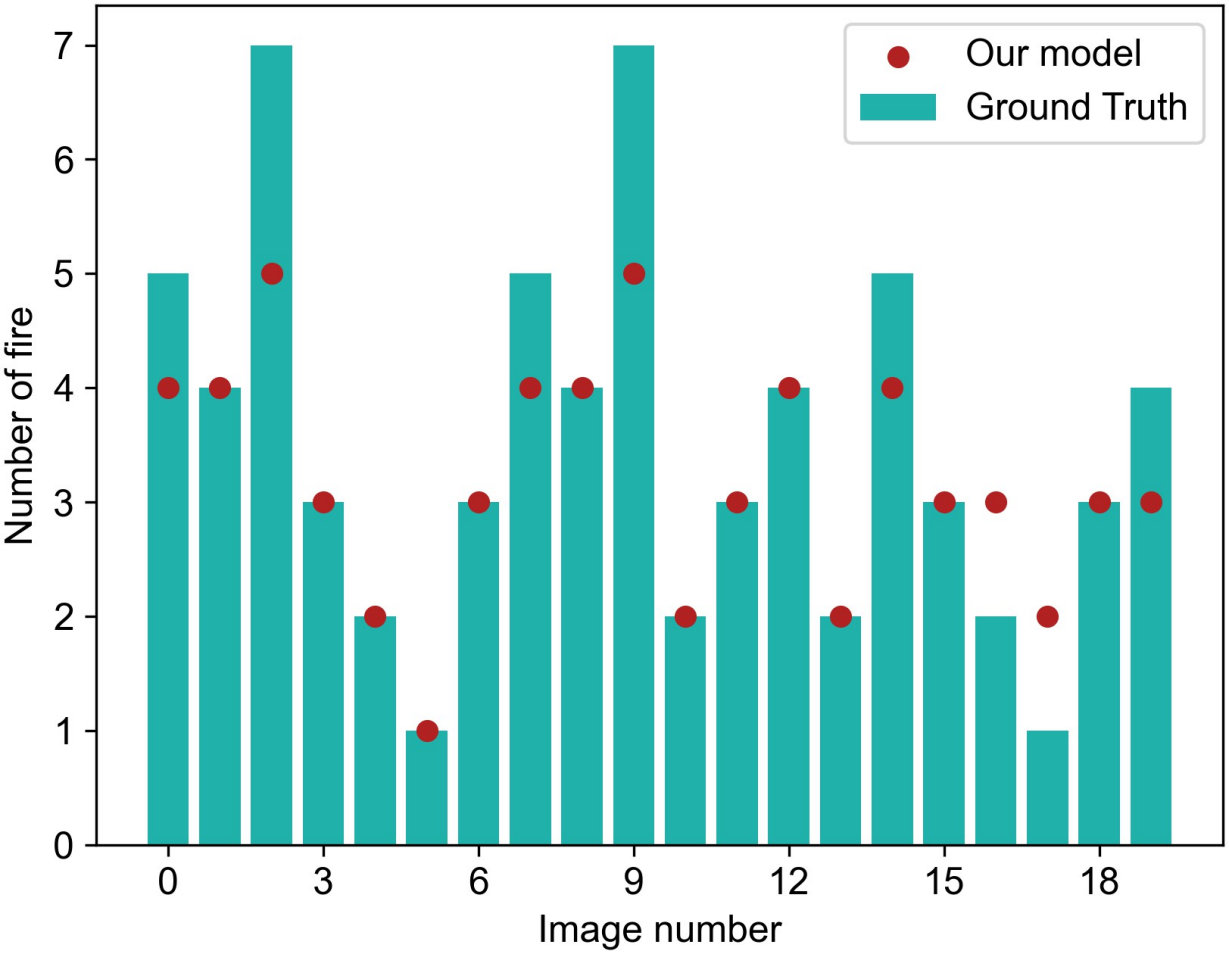
Comparison between actual value and the detected value.

When a fire starts, there will be many small flames, and the distribution may be more scattered, especially in coal mine, so that the model’s ability to detect is faced with a certain challenge. If the number of small flames can be counted, it will provide convenience for the firefighters’ rescue preparation. Therefore, we selected twenty images from the test set that contain many small flames to test the model for counting statistics. The comparison between detected results and the real number of flames are shown in Fig 10. In the figure, ground truth represents the actual number of flames. The data corresponding to the points in the figure represents the number of flame targets detected by our model, and the data corresponding to the columns represents the actual number of flame targets in the selected pictures. In this test, our model missed a total of eight targets and misdetected two. The actual number of targets in the image is 70, and a total of 64 targets have been detected. The average deviation between the ground truth and the predicted value is 0.6, which means that there may be only one false positive or false negative target for every two detections when there are many targets.

**Fig 11:**
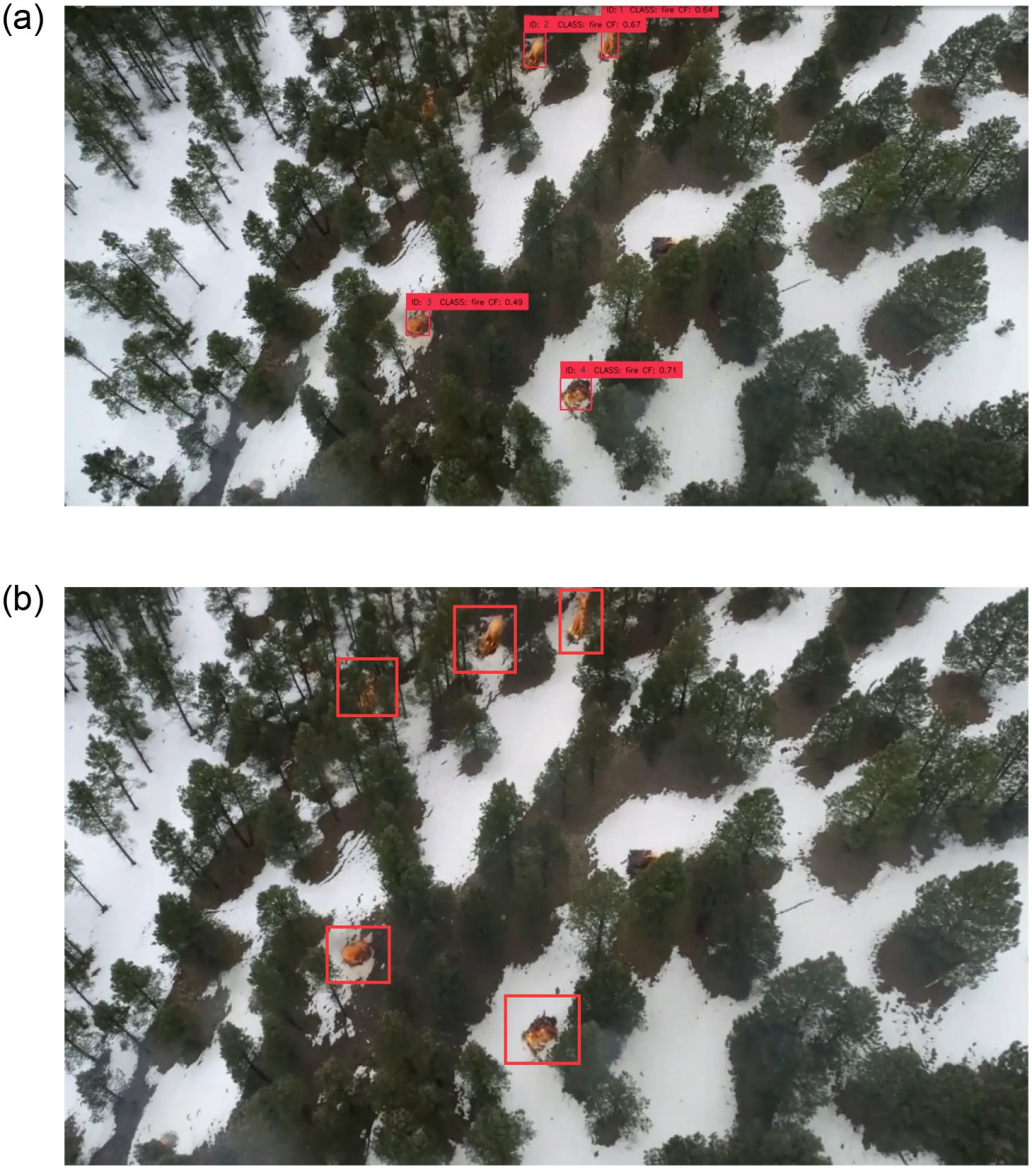
The comparison between the ground truth and the detection of our method. a: Our method; b: Ground Truth.

The counting results of one image in the dataset are shown in Fig 11. The ID in the figure is the number of each fire-smoke target, and the results are compared with the images marked by human. The robustness of the model suggested in this study is readily evident, as it yields relatively accurate counting results, even if the fire-smoke targets are small and distributed more scattered. In addition, it was found that the enhanced YOLOv8s have the capability to detect flames even in challenging environments such as snow forests.

### Effectiveness of detecting smoke

**Fig 12:**
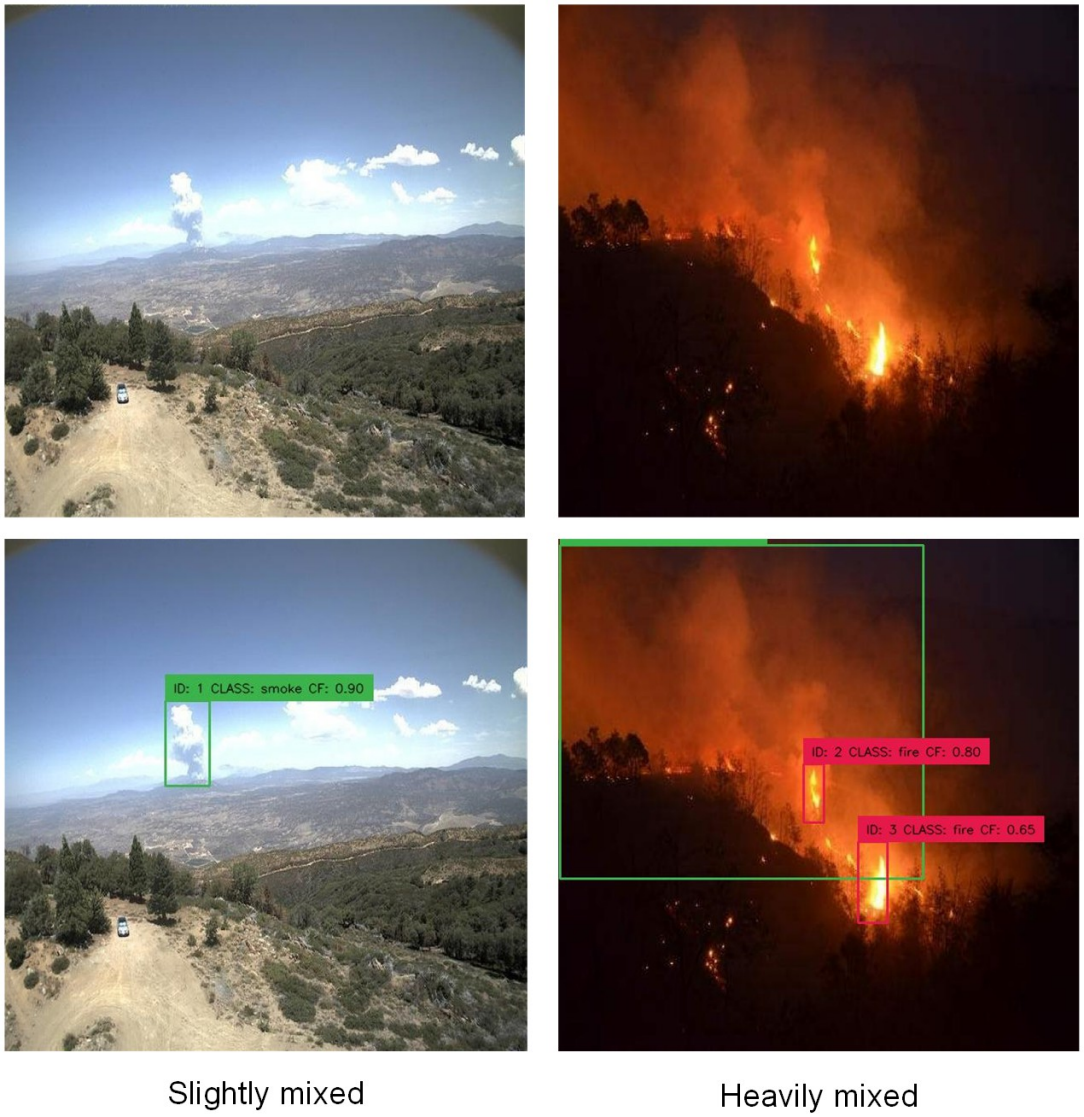
Comparison of the detection outcomes for smoke with various levels of background mixing.

The smoke detection accuracy of the model will be affected by the complex background in the coal mine environment. During the day, distant smoke may mix with similar white clouds, affecting the detection range of the model. At night, smoke will mix with the black background, and the outline is not clear enough, which may cause missed detection. In order to evaluate the model’s ability to detect objects in intricate settings resembling coal mines, which is the focus of this study, 20 images with high degree of smoke and background mixing were selected as dataset A, and 20 images with low degree of smoke and background mixing were selected as dataset B. The level of mixing serves as an independent variable, while the model was employed to detect datasets A, B, and A+B in sequential order. The detection results are shown in Table 5 and Fig 12. For smoke detection with low degree of background mixing (dataset A), mAP can reach 90.3%. Additionally, in environments with more complex backgrounds (dataset B), the model has the capability to attain a mAP value of 88.7%. When the two datasets are mixed into one dataset (A+B), the mAP of the model reaches 89.6%. The findings indicate that our model is successful in identifying smoke in various settings.

**Table 5:** Assessment indicators of smoke detection.

Test set	Precision	Recall	mAP	F1-score
*A*	93.3%	82.1%	90.3%	0.873
*B*	91.5%	81.2%	88.7%	0.860
*A* + *B*	92.3%	82.4%	89.6%	0.871

### Performance in varied environmental conditions

**Fig 13:**
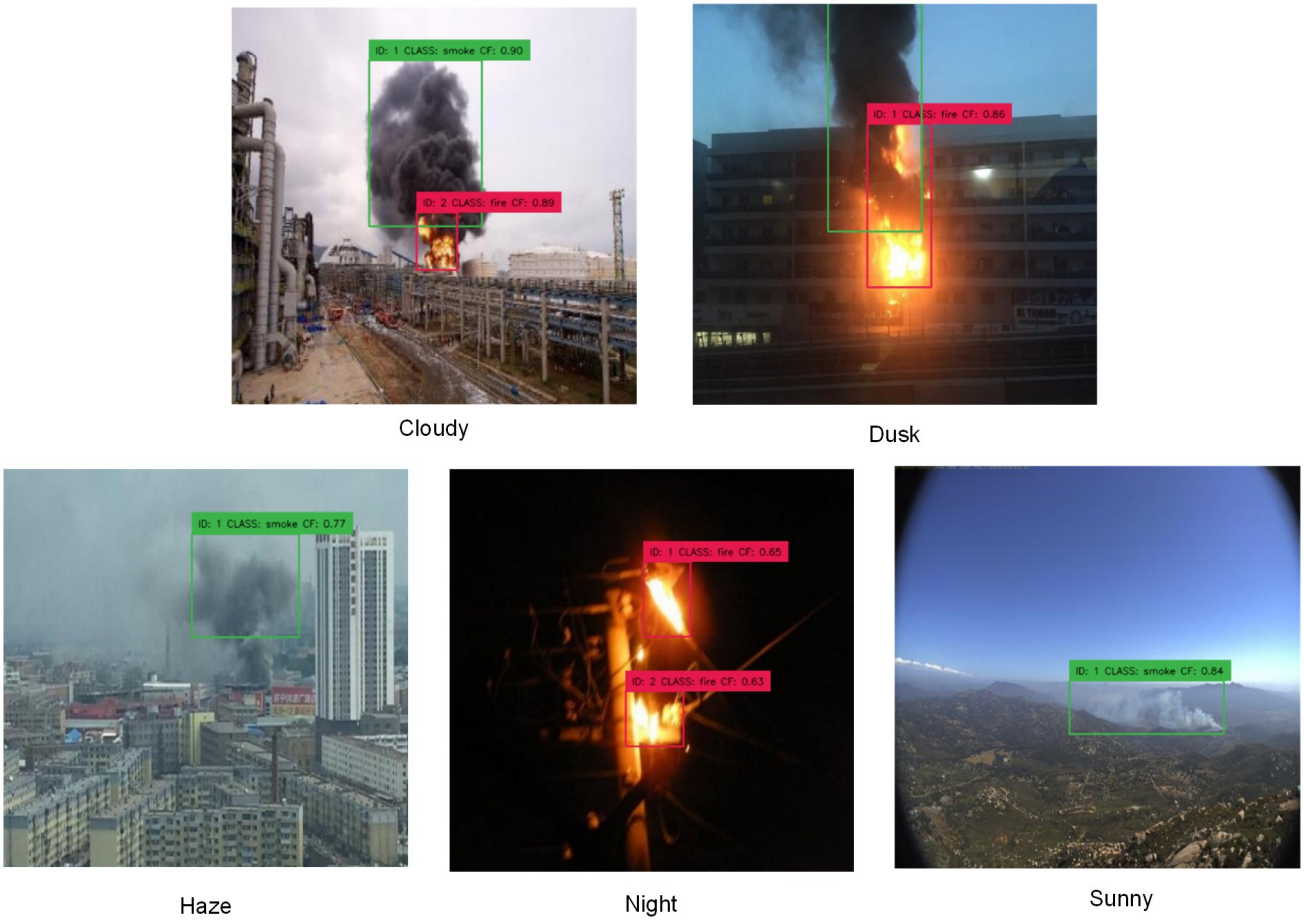
Performance in varied environmental conditions.

In order to achieve real-time detection tasks in coal mine environments, the model must exhibit high accuracy in detecting smoke and flames across various environmental conditions. Therefore, we selected ten images of smoke and flame for each of the five conditions: night, dusk, cloudy, haze, and sunny. These images were used to evaluate the performance of the model in different environments. The detection results are shown in Table 6 and Fig 13. The mAP of our model still can reach around 90 percent under challenging conditions such as cloudy weather and low-light environments. In the figure, we can see that under different brightness and background noise conditions, our model can still locate the target position accurately.

**Table 6:** Assessment indicators of detection in varied conditions.

Test set	Precision	Recall	mAP	F1-score
*night*	90.8%	84.1%	90.3%	0.873
*dusk*	89.9%	83.7%	89.6%	0.866
*cloudy*	89.3%	84.5%	89.9%	0.868
*haze*	88.1%	83.8%	88.1%	0.859
*sunny*	89.2%	82.2%	88.4%	0.856

### Ablation experiments

In order to evaluate the efficacy of each enhanced module, we performed a series of five ablation experiments and trained them in turn under the same experimental environment as before, and took the best weight files output by each training to perform experiments on the validation set. The epoch of each round of experiments was set to 300, and the experimental data obtained are shown in Table 7. After replacing the neck with Slim-neck, the mAP has decreased by 0.3 percentage points, but the Params, FLOPs, and Time have all decreased to varying degrees, which means that the model is lighter in complexity; after introducing PConv in the C2f module, the mAP decreased by 0.7 percentage points, but the Params, FLOPs, and Time (single image prediction time) have further decreased, leaving enough room for subsequent improvement; after adding the EMA module, the Params and FLOPs have slightly increased, but the mAP has increased by 1.9%, so it can be considered that this improvement point plays a crucial role in enhancing the identification of miniature objects; after replacing the original detection head with DyHead, the mAP increased by 1.6%, and at the same time, because the improvement introduced by DyHead only targets a certain dimension, Params and FLOPs are lower than before. The final model’s Time is 10.0ms, which has a slight increase, but still meets the requirements of real-time performance. In summary, compared with YOLOv8s, our model reduces the Params and FLOPs by 23% and 26%, respectively, while increasing the mAP by 2.5%. As can be seen, we have made significant progress in model light-weighting, which will make the application of object detection more widespread in coal mines.

**Table 7:** Different algorithms for smoke and fire detection.

Model	mAP	Time(ms)	Params(Million)	FLOPs(Giga)
*YOLOv*8*s*	88.5%	8.2	11.13	28.4
*YOLOv*8*s* + *Slim* − *neck*	88.2%	8.3	10.27	25.1
*YOLOv*8*s* + *Slim* − *neck* + *C*2*f* − *faster*	87.5%	7.6	8.82	20.9
*YOLOv*8*s* + *Slim* − *neck* + *C*2*f* − *faster* + *EMA*	89.4%	8.8	8.84	21.2
*YOLOv*8*s* + *Slim* − *neck* + *C*2*f* − *faster* + *EMA* + *DyHead*	91.0%	10.0	8.56	20.9

**Fig 14:**
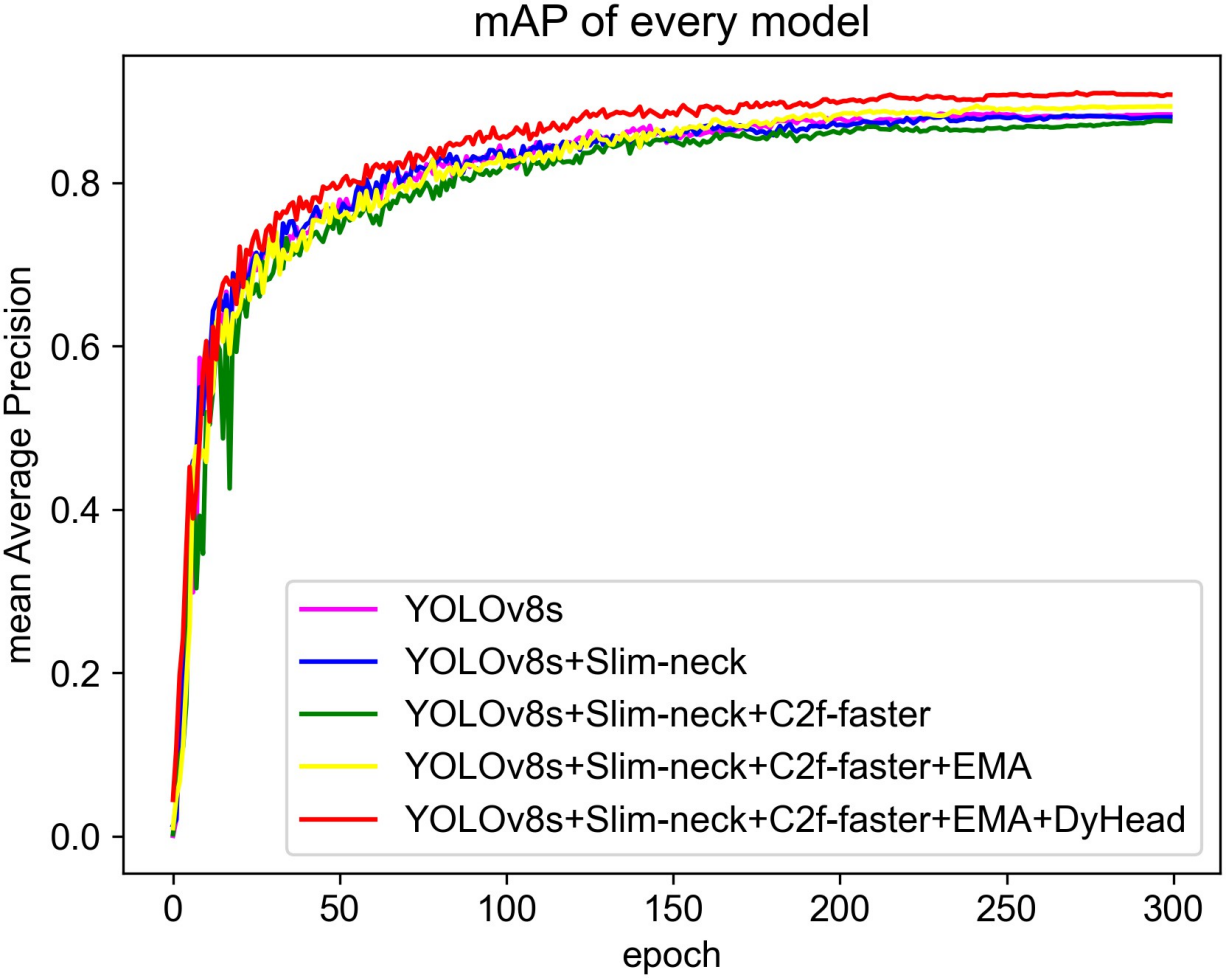
Comparison diagram of mAP.

In order to visually demonstrate the enhanced performance of the model, the mAP curves of the five models depicted in Fig 14. As can be seen, our model achieves a significantly higher mAP value than other models after about 50 epochs. This suggests that our model possesses a superior capacity to identify and classify fire and smoke present in the images, and can achieve a higher Precision and Recall. Therefore, our model is more effective and robust than other models for fire-smoke detection tasks.

## Conclusion

Fire-smoke detection is a very important part of coal mine safety. This paper proposes a deep learning-based real-time fire-smoke detection algorithm for coal mines, which improves the YOLOv8s network structure, making the model more lightweight, and enhancing its robustness and accuracy. The conclusions can be summarized as follows:

- We integrated, cropped, scaled, and rotated the images of two public fire-smoke datasets. This has expanded the dataset, and improved the anti-interference ability of the model after training.
- We chose YOLOv8s as the baseline model, which primarily comprises of three components: backbone, neck, and head. We improved each part of the baseline model, including using GSConv and VoV-GSCSP to lighten the neck part, using PConv to lighten the C2f module, introducing the EMA mechanism to enhance the backbone part’s detection ability for small targets, and using DyHead to enhance the head part’s representation ability.
- Our model can effectively detect smoke and flame targets under different environmental conditions. Compared with some classic object detection models, our model is more suitable for coal mine, because we has made progress in terms of accuracy, and computation complexity.

In the coal mine, the model we proposed mainly has the following two application scenarios. Since our model is less complex, the deployment process will be easier.

- Embedded in the UAV for smoke-fire monitoring: Current UAV can already automatic takeoff, charge, and patrol according to a preset path. Therefore, UAV could be used for routine fire monitoring with our model.
- Embedded in fixed cameras for smoke-fire monitoring: Fixed position cameras can be set up in high fire risk locations in coal mines, and their returned images can be monitored by our model.

The improved YOLOv8s object detection model will provide a more applicable idea for real-time fire-smoke detection in coal mines. But in this study, we didn’t fully explore the working effect of our model under other types of coal mines. In future work, we will focus on studying and solving the scalability and adaptability of YOLOv8 in different types of coal mines. We believe that this will make a great contribution to the intelligent process of coal mine operation.
